# Mycobacterial FtsEX-RipC interaction is required for normal growth and cell morphology in rifampicin and low ionic strength conditions

**DOI:** 10.1128/spectrum.02515-23

**Published:** 2024-01-30

**Authors:** Veneshley Samuels, Andani E. Mulelu, Hlumani Ndlovu, Mohlopheni J. Marakalala

**Affiliations:** 1 Division of Medical Microbiology, Department of Pathology and Institute of Infectious Disease and Molecular Medicine, Faculty of Health Sciences, University of Cape Town, Cape Town, South Africa; 2 Division of Medical Biochemistry and Structural Biology, Department of Integrative Biomedical Sciences, Faculty of Health Sciences, University of Cape Town, Cape Town, South Africa; 3 Division of Chemical Systems Biology, Department of Integrative Biomedical Sciences and Institute of Infectious Disease and Molecular Medicine, Faculty of Health Sciences, University of Cape Town, Cape Town, South Africa; 4 Division of Immunology, Department of Pathology and Institute of Infectious Disease and Molecular Medicine, Faculty of Health Sciences, University of Cape Town, Cape Town, South Africa; 5 Africa Health Research Institute, Durban, KwaZulu-Natal, South Africa; 6 Division of Infection and Immunity, University College London, London, United Kingdom; University of Warwick, England

**Keywords:** FtsE, FtsX, FtsEX, RipC, *Mycobacterium tuberculosis*, *Mycobacterium smegmatis*, cell wall

## Abstract

Tuberculosis, a lung disease caused by *Mycobacterium tuberculosis* (*Mtb*), remains a major global health problem ranking as the second leading cause of death from a single infectious agent. One of the major factors contributing toward *Mtb’s* success as a pathogen is its unique cell wall and its ability to counteract various arms of the host’s immune response. A recent genome-scale study profiled a list of candidate genes that are predicted to be essential for *Mtb* survival of host-mediated responses. One candidate was FtsEX, a protein complex composed of an ATP-binding domain, FtsE, and a transmembrane domain, FtsX. FtsEX functions through interaction with a periplasmic hydrolase, RipC. Homologs of FtsEX exist in other bacteria and have been linked with playing a key role in regulating peptidoglycan hydrolysis during cell elongation and division. Here, we report on *Mycobacterium smegmatis,* FtsE, FtsX, and RipC and their protective roles in stressful conditions. We demonstrate that the individual genes of FtsEX complex and RipC are not essential for survival in normal growth conditions but conditionally essential in low-salt media and antibiotic-treated media. Growth defects in these conditions were characterized by short and bulgy cells as well as elongated filamentous cells. Our results suggest that FtsE, FtsX, and RipC are required for both normal cell elongation and division and ultimately for survival in stressful conditions.

**IMPORTANCE:**

Mycobacterial cell growth and division are coordinated with regulated peptidoglycan hydrolysis. Understanding cell wall gene complexes that govern normal cell division and elongation will aid in the development of tools to disarm the ability of mycobacteria to survive immune-like and antibiotic stresses. We combined genetic analyses and scanning electron microscopy to analyze morphological changes of mycobacterial FtsEX and RipC mutants in stressful conditions. We demonstrate that FtsE, FtsX, FtsEX, and RipC are conditionally required for the survival of *Mycobacterium smegmatis* during rifampicin treatment and in low-salt conditions. Growth defects in these conditions were characterized by short and bulgy cells as well as elongated filamentous cells. We also show that the FtsEX-RipC interaction is essential for the survival of *M. smegmatis* in rifampicin. Our results suggest that FtsE, FtsX, and RipC are required for normal cell wall regulation and ultimately for survival in stressful conditions.

## INTRODUCTION

Tuberculosis (TB) remains a major global health challenge causing 1.6 million deaths annually (1). TB disproportionately affects the most vulnerable strata of society, which is highlighted by the most severe burden of the disease in sub-Saharan Africa (2). The success of *Mtb* as a pathogen lies in its ability to survive within the host cells and to evade the killing capacity of the immune system (3). Alveolar macrophages form the first line of defense in eliminating *Mtb* and are armed with several immune effector mechanisms capable of both detecting and combating the invading pathogen (4, 5). Macrophages employ a potent antimicrobial arsenal, which includes phagosome maturation, phagolysosome fusion as a killing mechanism via acidification, production of reactive oxygen and nitrogen intermediates, and nutrient starvation (6). While all these effector mechanisms pose a threat to the intracellular survival of *Mtb*, mycobacteria have been shown to express genes that prevent phagosome maturation events and subvert the killing effect of the macrophages (6).

A key trait of *Mtb* is its unique and thick cell wall, which not only maintains optimal homeostasis but also protects the bacterium from harsh environmental threats (7, 8). Cell wall homeostasis is, therefore, crucial and requires tight regulation that is maintained by a complex of genes that is key for successful growth and division, especially when bacteria are exposed to stressful conditions (7, 9, 10). Careful breakdown of the mycobacterial complex cell wall is regulated by several protein complexes and hydrolases, particularly amidases, forming the hydrolytic pathways that enable faithful sharing of cell wall material between daughter cells (11, 12). A recent study has demonstrated the requirement of a mycobacterial amidase, ami1, in the maintenance of normal cell division. The deletion of ami1 also resulted in susceptibility to cell wall-targeting antibiotics and increased cell wall permeability (13). It is still not yet fully clear where is the location of cell elongation and division, and how signals triggered to drive these processes are controlled. Our understanding of these events is based on the remodeling of the peptidoglycan (PG) layer, which becomes hydrolyzed at the correct time and place, allowing daughter cells to separate (11). How mycobacteria ensure that PG hydrolases function only in the correct spatial and temporal context remains largely unknown. Unchecked hydrolysis can cause lysis of the cells, while deactivated hydrolysis may result in the formation of chaining cells (14–16).

An emerging model is that mycobacterial FtsEX-RipC forms a complex, which senses the progress of division and regulates extracellular PG hydrolases (11). Mycobacterial FtsEX-RipC is a combination of a cytoplasmic ATPase, FtsE, a transmembrane protein, FtsX, and a periplasmic PG hydrolase, RipC (11, 17–19). Homologs of the FtsEX complex exist in other bacteria, including *Escherichia coli*, *Streptococcus pneumoniae*, *Caulobacter crescentus,* and *Bacillus subtilis*, where it has been reported to play a role in events related to the regulation of PG hydrolysis during cell elongation and division (11, 17, 20–22). In *Mtb*, FtsEX has been implicated as an important contributor to the pathogen’s evasion of CD4-mediated immune responses in a genome-wide transposon site hybridization approach (9). Based on this, we postulated that mycobacterial FtsEX-RipC domains are involved in the regulation of cellular elongation and division, particularly when the bacterium is exposed to host immune-related stresses and antibiotic stress. *Mtb FtsX* shares 76% sequence homology with *M. smegmatis FtsX*, while there is 82% sequence homology between *Mtb* and *M. smgematis FtsE*. The high sequence similarity in the *FtsE* and *FtsX* between the two organisms makes it feasible to utilize *M. smegmatis* as a model to study the function of the mycobacterial FtsEX complex genes in response to stress. Therefore, in this study, we investigated the biological essentiality of *M. smegmatis* FtsE, FtsX, and RipC in stressful conditions, including low osmotic and antibiotic stress. We utilize scanning electron microscopy to demonstrate the requirement of the individual genes in the maintenance of normal cell morphology under stressful conditions.

## MATERIALS AND METHODS

### Construction of *M. smegmatis* knockout strains

About 500 bp of upstream and downstream flanking regions of the genes *ftsE*, *ftsX*, *ftsEX,* and *ripC* were amplified from the genomic DNA of *Mycobacterium smegmatis* MC^2^155 using Gotaq DNA polymerase (Promega, USA) PCR approach. The specific target genes were inactivated by replacement of a hygromycin resistance cassette using overlap extension PCR. The resultant constructs were electroporated into an acetamide-inducible *M. smegmatis* MC^2^155 pJV53 recombinase strain. Mutant strains were selected on 7H10 Middlebrook agar plates containing 50 µg/mL hygromycin and 25 µg/mL kanamycin. Specific regions within all the strains were sequenced to confirm the location of the inserts.

### Complementation of strains

Knockout and complement strains used in the study are listed in Table 1. The strains were constructed according to Mavrici et al. (11). FtsEX was PCR-amplified from *M. smegmatis MC^2^ 155* genomic DNA, with 5′ and 3′ prime ends containing unique restriction sites for BspH1 and HindIII, respectively. Digested PCR products were ligated into a predigested pDE43-MCzq1, a zeocin-marked destination vector that integrates at L5 site of the genome. The pDE43-MCzq1 plasmid containing the correct insert was confirmed by DNA sequencing and is referred to here as pFtsEX. pFtsEX was transformed into ΔFtsEX strain and plated on agar containing 50 µg/mL hygromycin and 20 µg/mL zeocin. To construct point mutants in the FtsEX extracellular domain (ECD) known to interact with the hydrolase (11).

**TABLE 1:** *Mycobacterium smegmatis* strains used in this study

Simplified name	Full cloning strategy
ΔFtsX	ΔFtsX::HygR
ΔFtsE	ΔFtsE::HygR
ΔRipC	ΔRipC::HygR
ΔFtsEX	ΔFtsEX::HygR
ΔFtsEX:pFtsEX	ΔFtsEX::HygR-L5::pDE-MCzq-FtsEX
ΔFtsEX:pFtsEXF61A	ΔFtsEX::HygR-L5::pDE-MCzq-FtsEXF61A
ΔFtsEX:pFtsEXF110A	ΔFtsEX::HygR-L5::pDE-MCzq-FtsEXF110A
ΔFtsEX:pFtsEXY113A	ΔFtsEX::HygR-L5::pDE-MCzq-FtsEXY113A
ΔFtsEX:pFtsEXF122A	ΔFtsEX::HygR-L5::pDE-MCzq-FtsEXF122A

### Bacterial growth analysis

*M. smegmatis* strains were maintained on Middlebrook 7H10 agar and 7H9 media containing 0.2% glycerol and supplemented with 10% albumin dextrose catalase (ADC) and 0.05% Tween 80 (complete media). Strains were grown in media containing various stressors. For growth analysis, bacteria were grown from a starting optical density (OD) of 0.001 in normal complete 7H9 media, 0% NaCl LB media, or 7H9 media containing 1 mM rifampicin. Bacteria were incubated with continuous shaking at 37°C. Absorbance (600 nm) was read using standard methods every 12 hours for 48 hours. Data were plotted and analyzed using GraphPad Prism 6.

### Bacterial colony-forming unit (CFU) determination

Strains were grown from a starting OD of 0.001 in 7H9 medium containing 0.2% glycerol and supplemented with 10% ADC and 0.05% Tween 80 with or without 1 µg/mL rifampicin. At 0 and 36 hours, the bacterial cultures were plated with serial dilutions on 7H10 agar plates. Plates were incubated at 37°C and colonies were enumerated after 3 days. Data were plotted and analyzed using GraphPad Prism 6.

### Scanning electron microscopy

Samples were prepared according to the SEM protocol of the Electron Microscope Unit at the Centre of Imaging (University of Cape Town, Division of Chemical Engineering, Upper Campus). The *M. smegmatis* mutants were exposed to the different stressful media conditions and samples were taken at log and stationary growth phases. After centrifugation at 4,000 rpm for 30–60 seconds, samples were fixed with 2.5% (vol/vol) glutaraldehyde for approximately 8 hours in a 4°C fridge. 1× Phosphate-buffered saline (PBS) was used to rinse the samples before fixing them with 1% (mass/vol) osmium tetroxide for approximately 1 hour. Cells were spun and washed with 1× PBS and dH_2_O to remove any excess osmium. Following a series of dehydrations in 30%, 50%, 70%, 90%, 95%, and 100% ethanol, cells were resuspended in 100% ethanol and mounted on microscope aluminum stubs. On the stubs, cells were dried by gently adding two to three droplets of hexamethyldisilazane reagent (Sigma-Aldrich) and sputter coated with carbon. After 20-minute incubation at room temperature, samples were placed in the scanning electron microscope (SEM-MIRA) and images were recorded and analyzed using the MIRA3 TESCAN program.

### Microscopy

Bacterial strains (starting OD600 = 0.03) were grown for 12 hours with shaking at 37°C. The cells were labeled with FM4-64 dye (Invitrogen) and visualized (Nikon Eclipse). Cell length was calculated using ImageJ software (National Institutes of Health) and converted into microns or nanometres using the appropriate pixel to micron/nanometre conversion. Length was measured from the cell pole to the opposite cell pole.

## RESULTS

### FtsE, FtsX, and RipC are required for *M. smegmatis* survival in various stressful conditions

**Fig 1:**
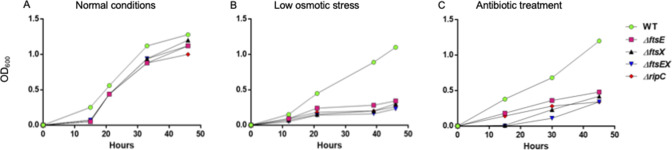
ΔFtsE, ΔFtsX, ΔFtsEX, and ΔRipC have impaired growth in rifampicin and low-salt conditions. (**A**) Growth curves of wild-type MC^2^155 *M. smegmatis*, FtsE, FtsX, FtsEX, and RipC mutants in normal 7H9 media, (**B**) low osmotic media, and (**C**) 7H9 media containing 1 µg/mL rifampicin drug. The graphs are representative of three biological replicates.

Genes that govern normal elongation and division are likely to be essential for bacterial survival. To investigate whether the loss of FtsEX-RipC-interacting complex compromises the ability of *M. smegmatis* to grow normally, we constructed *ftsX*, *ftsE*, *ripC,* and *ftsEX* knockout strains and studied the biological essentiality of these genes under *in vitro* stressful conditions. The mutants exhibited a delayed growth of 15 hours before resuming normal growth dynamics compared to the wild type (WT) (Fig. 1A). The subsequent growth rate observed between the WT and mutants was comparable between 20 and 48 hours, suggesting that FtsEX-RipC is not essential for growth in normal conditions.

Pioneering work by Ricard and Hirota has previously located *ftsE* as a gene involved in cell division, particularly in *E. coli (23, 24*). One important property associated with FtsE in *E. coil* is that it is salt remedial, which means that cell viability is restored upon the inclusion of salt (NaCl) in the growth medium (24). To study the effect of salt-depleted media on bacterial growth and morphology, all strains were exposed to low osmotic conditions created by depleting LB broth of NaCl (0% NaCl) . All mutants followed a similar poor growth trend, reaching a peak OD_600_ of about 0.3, whereas the WT strain thrived reaching a peak OD_600_ of 1.1 (Fig. 1B). This suggested that FtsE and FtsX, the individual genes of the FtsEX-RipC complex, are required for growth in low osmotic conditions.

We next exposed WT, ΔFtsE, ΔFtsX, ΔFtsEX, and ΔRipC to rifampicin treatment after determining the concentrations using the Alamar Blue MIC assay (Table S2). In the rifampicin-treated media, ΔFtsX and ΔftsEX experienced a suppressed lag phase of 15 hours, indicated by the undetectable bacterial growth (Fig. 1C). The WT grew effortlessly reaching a peak OD_600_ of 1.3 at 48 hours as opposed to all mutant strains (Fig. 1C). *Δ*FtsX and *Δ*FtsEX displayed a delayed growth, reaching the OD_600_ of less than 0.4, while ΔFtsE and ΔRipC also showed low bacterial growth rates compared to the WT, suggesting that all mutant strains are sensitive to rifampicin treatment (Fig. 1C).

### FtsE, FtsE, and RipC mutants have morphological defects in rifampicin and low-salt conditions

**Fig 2:**
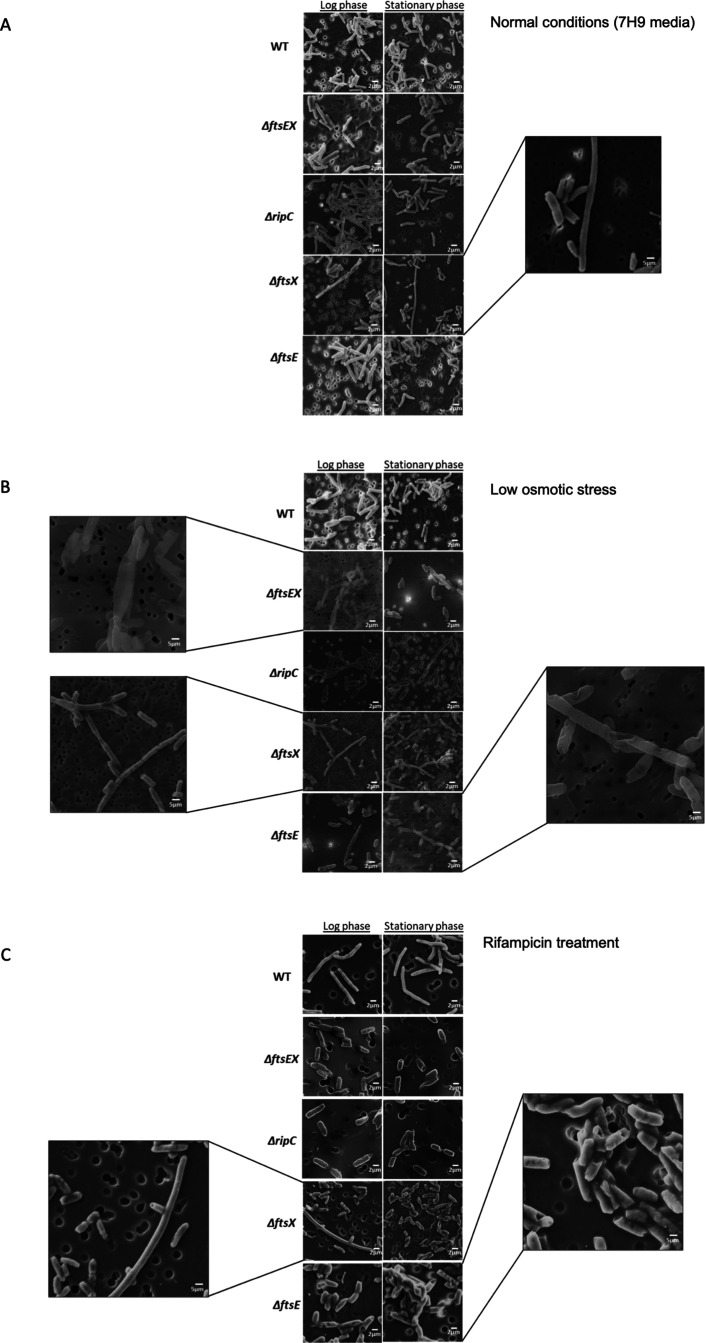
ΔFtsE, ΔFtsX, ΔFtsEX, and ΔRipC have morphological defects in stressful conditions. (**A**) Analysis of WT *MC^2^155 M. smegmatis*, ΔFtsE, ΔFtsX, ΔFtsEX, and ΔRipC under normal conditions. WT viewed at 20X magnification in log and stationary phases. ΔFtsE, 20X magnification in log and stationary phases. ΔFtsX 20X magnification in log and stationary phases. ΔFtsEX*,* 20X magnification in log and stationary phases. ΔRipC*,* 20X magnification in log and stationary phases. All strains were grown in duplicates. Scale bar = 2 µM. (**B**) Analysis of WT *MC2155 M. smegmatis*, ΔFtsE, ΔFtsX, ΔFtsEX, and ΔRipC in low osmotic media conditions. Salt-depleted LB broth was used to create low osmotic stress. WT 20X magnification in log and stationary phases. ΔFtsE, 20X magnification in log phase and stationary phase. ΔFtsX, 20X magnification in log phase and stationary phase. ΔFtsEX, 20X in log phase and stationary phase. ΔRipC*,* 20X magnification in log phase and stationary phase. Zoomed in region of the ΔFtsX indicates chaining and zoomed in ΔFtsEX region shows bulging. Strains were grown in duplicates. Scale bar ranges = 2 and 5 µM. (**C**) Analysis of WT *MC2155 M. smegmatis*, ΔFtsE, ΔFtsX, ΔFtsEX, and ΔRipC under antibiotic stress. Concentration of rifampicin drug was 1 µg/mL. WT viewed at 20X magnification in log and stationary phases. ΔFtsE, 20X magnification in log and stationary phases. ΔFtsX, 20X in log and stationary phases. ΔFtsEX, 20X magnification in log and stationary phases. ΔRipC*,* 20X magnification in log and stationary phases. Zoomed in region of ΔFtsE indicates the formation of holes at the edges of the cells and zoomed in region of ΔFtsX indicates unusual branching. Strains were grown in duplicates. Scale bar ranges = 2 and 5 µM.

**Fig 3:**
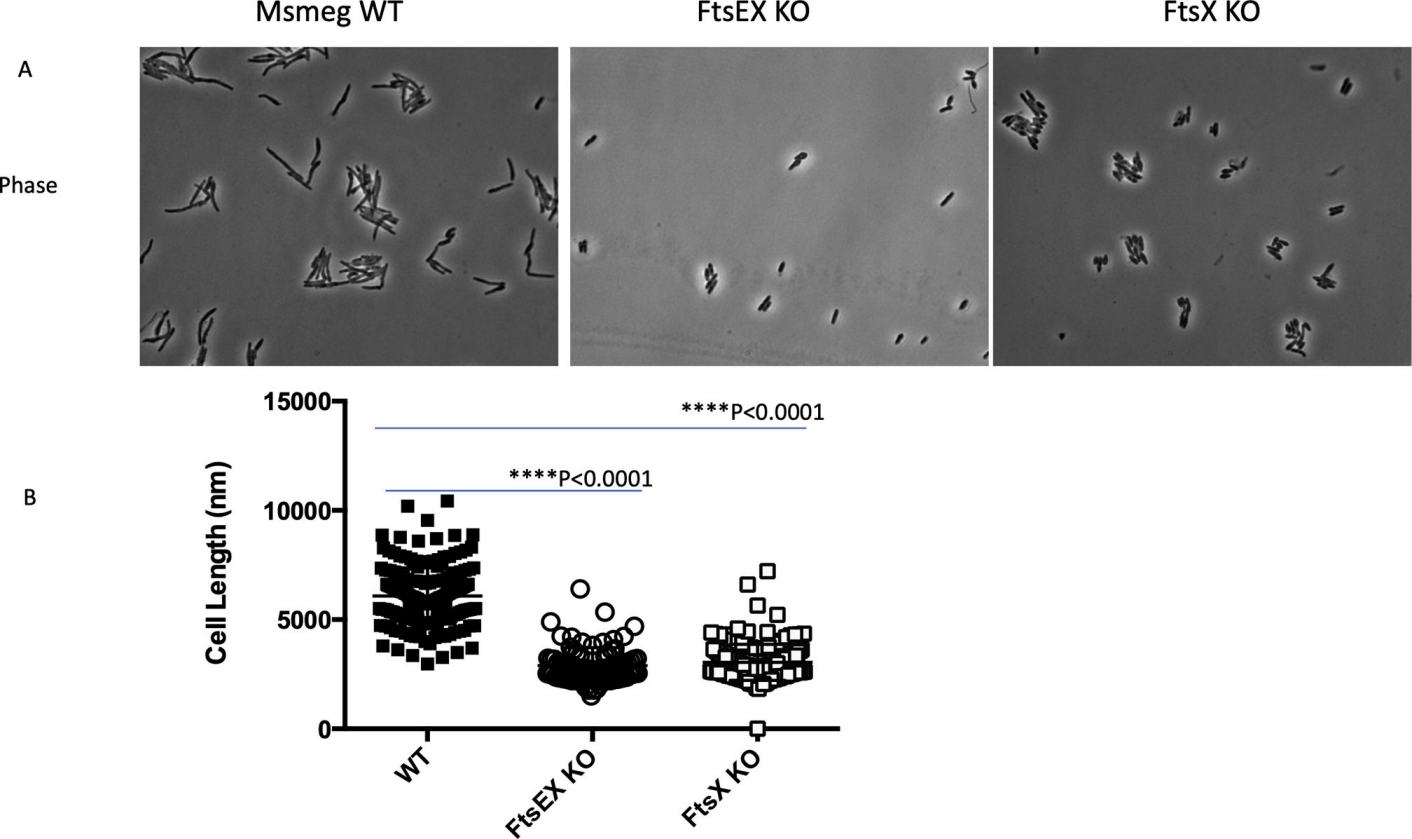
Deletion of *M. smegmatis, FtsEX,* and *FtsX* results in shorter cells. (**A**) Microscopy images of log phase cells of the ftsEX and fstX mutants in low osmotic strength. The images show that ftsEX and fstX mutants are shorter than WT cells. (**B**) Quantification of cell-length distribution of WT (125 cells), FtsEX KO (110), and FtsX KO (100) in log phase in low osmotic medium, indicating the requirement of *M. smegmatis* FtsEX in the maintenance of normal cell elongation under the stressful condition. Length was measured from cell pole to the opposite cell pole. Between 100 and 125 cells per field were counted. Data represent three biological replicates. Data were plotted and analyzed using GraphPad prism. Student’s *t*-test was used for statistical comparisons. ****P < 0.0001.

To characterize growth-related phenotypes of FtsEX and RipC mutants under various conditions, we performed growth phase-dependent imaging using scanning electron microscopy. Under normal conditions, all the mutants exhibited similar morphology to the wild type in all the growth phases except for FtsX mutants that displayed a filamentous or a usually short length at the log and stationary phases of growth (Fig. 2A; Fig. S1). Our results, therefore, indicate that FtsEX, RipC, FtsE, and FtsX are not essential for growth in normal conditions. Significant morphological changes were seen between the WT and mutant strains when cells were exposed to low osmotic stress (Fig. 2B). The exposure to the stress did not affect WT cells (Fig. 2B). In contrast, visible differences in cell length and morphology were seen in ΔFtsE, ΔFtsX, ΔFtsEX, and ΔRipC mutants. All mutants had a combination of short and long cells that were seen in log and stationary phases (Fig. 2B). Some of the cells that were shorter in length were bulgy, while those that were longer in length had a filamentous phenotype. Furthermore, the septum for some of the filamentous cells appeared to be invaginated, which was accompanied byh visible chaining, as was observed with ΔFtsE at the stationary phase. Another important observation is the extreme bulging that was exclusively observed for FtsEX mutants (Fig. 2B). To confirm these findings, we imaged more representative images on phase contrast microscopy and quantified cell lengths of FtsEX mutants and WT cells. FtsEX and FtsX mutants were significantly shorter than WT in low osmotic conditions (Fig. 3A and B). Our data indicate that deletion of the individual genes of the FtsEX-RipC complex results in growth defects accompanied by morphological abnormalities in low osmotic stress.

Some of the ΔFtsE and ΔFtsX shorter cells appeared to have holes in their cell wall when they were exposed to rifampicin (Fig. 2C, zoomed in ΔFtsE stationary phase). This phenotype was also observed for one or two ΔFtsEX cells (Fig. 2C). WT cells lacked this characteristic and appeared to be unaffected by the exposure to rifampicin. In the stationary phase, no filamentation was observed in ΔftsE, ΔFtsX, ΔFtsEX, and ΔRipC cells. Along with the altered cell lengths, the shorter cells in ΔFtsE, ΔFtsX, ΔFtsEX, and ΔripC looked fatter and bulgier (Fig. 2C; Fig. S1). At log phase, ΔFtsX had a distinct filamentous cell that was partially curved with unusual branching along the pole of the cell. These results suggest that the FtsE, FtsX, and RipC mutant cells fail to maintain shape during rifampicin treatment (Fig. 2C).

### FtsEX and RipC interaction is required for the survival of *M. smegmatis* in low osmotic stress and during rifampicin treatment

**Fig 4:**
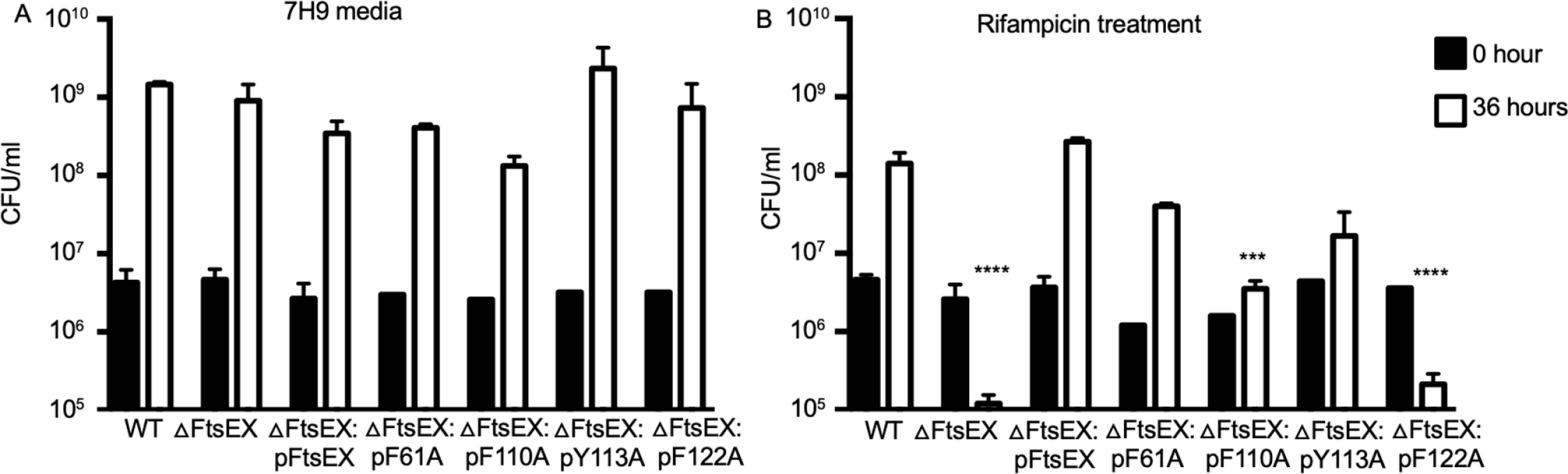
FtsX ECD point mutants are sensitive to rifampicin. ΔFtsEX mutant complemented with wild-type FtsEX and the indicated FtsX ECD allele were grown in 7H9 media with no antibiotic (**A**) and in 7H9 media containing 1 mg/mL rifampicin (**B**). CFU were plated at 0 and 36 hours. Values are the average of three biological replicates. Student’s *t* test was used to determine statistical differences between WT versus other strains treated with rifampicin at 36 hours. Error bars represent the SD from the mean. Data were plotted and analyzed using GraphPad Prism. ****P < 0.0001 and ***P < 0.001

We have previously shown that mycobacterial FtsX interacts with the hydrolase, RipC, through the ECD (11). To determine if the interaction was required for bacterial survival in the low osmotic stress conditions, we compared the growth of the FtsEX and RipC mutants with the FtsEX and RipC mutant strains complemented with the wild-type *FtsEX* and *RipC* genes, respectively. Compared to WT, both FtsEX and RipC mutants phenocopied each other with the reduction in growth (Fig. S2). The growth defects were reversed by complementation, confirming the requirement of FtsEX and its interacting hydrolase, RipC, for survival in low-salt conditions. To determine if the interaction was required for bacterial survival during rifampicin treatment, we compared the growth of the FtsEX mutant with the FtsEX mutant strain complemented with the wild-type FtsEX gene or with the FtsX ECD point mutants (FtsX F61A, F110A, Y113A, and F122A) that we previously identified (11). All the *M. smegmatis* mutants and the complemented strains grew normally in 7H9 normal media, with the colony count of just over 1 × 10^6^ CFU/mL and about 1 × 10^9^ CFU/mL at 0 and 36 hours, respectively (Fig. 4A). However, when treated with rifampicin, the FtsEX mutant was hypersensitive to the drug with the colony count reduced to 1 × 10^5^ CFU/mL after 36 hours. The FtsEX mutant complemented with wild-type *ftsEX* restored growth to the levels of the WT cells (1 × 10^8^ CFU/mL). However, the FtsEX mutant cells complemented with FtsX F61A, F110A, Y113A, and F122A mutants were hypersensitive to rifampicin (Fig. 4B). The F122A mutation was the most severe, resembling the *ftsEX* deletion (blocking growth about 10-fold). The F110A and Y113A substitutions resulted in partial impairment of bacterial growth. These results indicate that RipC-binding residues in the FtsX ECD are crucial for function and that the FtsEX interaction with RipC is required for mycobacterial growth during rifampicin treatment.

## DISCUSSION

Cell division is a fundamental process in the life cycle of all bacteria (25). The entire process must be carefully coordinated and requires tight regulation in response to environmental changes (11). Bacterial proliferation is divided into two main processes: elongation and division. Large multiprotein complexes known as the elongation complex and divisome help maintain a constant balance between cell wall degradation and synthesis. When a bacterial cell divides, it enlarges its peptidoglycan layer and subsequently divides it too. Hence, PG remodeling is important for bacterial growth and depends on the modulating activities of these complexes (11, 25).

PG hydrolases play a key role in coordinating the breakdown of PG. The exact time and space are important for this coordination because they enable faithful sharing of the cell wall material to facilitate daughter cell separation. We have previously shown the importance of FtsEX as a gene complex in response to stressful conditions. However, the role of the individual proteins, FtsE and FtsX, is still poorly understood. Utilizing scanning electron microscopy, in the current work, we characterized FtsE and FtsX growth and morphology in rifampicin and low-salt conditions. We focused on the mycobacterial cell wall proteins, FtsX and FtsE, as well as their interacting periplasmic PG hydrolase, RipC. Homologs of FtsEX exist in distantly related bacterial species, and consistent trends of evidence link it to a key role in the regulation of PG hydrolysis during elongation and division. Little is known about FtsE, FtsX, and RipC in growth and division, particularly of their role in the context of immune-related stressors. Hence, we investigated the biological essentiality of *M. smegmatis,* FtsE, FtsX, and RipC in stressful conditions that mimic the immune-related conditions and antibiotic stress *in vivo*.

We successfully generated FtsEX, FtsE, FtsX, and RipC mutant strains by knocking out the gene of interest using a 2200 bp product that contained the upstream and downstream flanking regions stitched to a HygR cassette. We and others have consistently used the gene knockout method with the hygromycin cassette to successfully generate specific gene knockouts without causing a frameshift in the downstream genes (11). We then determined the sensitivity of FtsEX and RipC mutants to anti-TB drugs using Alamar blue MIC determination assays. Subsequently, we selected rifampicin as an antibiotic condition to be examined in our growth curve conditions. We evaluated the growth dynamics of the mutants and wild type in media lacking essential minerals or supplemented with harmful molecules to mimic the harsh environment bacteria encounter inside macrophages. All the mutants and wild-type bacteria exhibited similar growth dynamics in normal 7H9 media, suggesting that ΔFtsE, ΔFtsX, ΔFtsEX, and ΔRipC are not essential for survival in normal conditions. Conversely, growth was severely altered in the absence of salt/salt-depleted media, which is similar to what was observed for FtsEX in *E. coli (26*).

We found that rifampicin was able to restrict the growth of all the mutant strains. The complementation of FtsEX with the native gene sequence fully reversed the phenotypes in both low-salt media and during rifampicin treatment, confirming that the phenotypes observed were due to the gene deletion. Mavrici and colleagues showed that sublethal concentrations of rifampicin severely inhibit the growth of FtsEX and RipC mutants (11). Rifampicin is known to inhibit transcription by targeting RNA polymerase (rpoB) (27). On that account, the hypersensitivity of the mutants to rifampicin could be due to the need for efficient transcription of a functionally redundant system. The sensitivity of the mutants could also mean that drugs are accumulating at higher levels than the WT due to changes in the cell wall. A similar effect has been previously shown by Senzani et al., in which the deletion of mycobacterial ami1 resulted in increased cell wall permeability (13).

Depletion of FtsEX generates cell morphology defects characterized by filamentous cells that are completely assembled but not separated (28). We decided to do growth phase-dependent imaging to look for cell division-specific characteristics such as defects in elongation and division. Consistent with their nonessential role in normal conditions, FtsE, FtsEX, and RipC mutants had similar morphological phenotypes with the WT strain. The deletion of *ftsX*, however, resulted in the formation of a heterogeneous population comprising shorter or lengthier cells in normal conditions. However, the morphological changes were minor, suggesting that FtsX may not play a major cell elongation/division role in normal conditions. Since FtsE directly interacts with FtsX, we had expected the deletion of *ftsE* to phenocopy the same cell defects caused by the deletion of *ftsX*; however, this was not the case, particularly in normal conditions. This finding contrasts with that observed for FtsE and FtsX in *S. pneumoniae (29*). Interestingly, the resulting cell division defects were not correlated with a reduced growth rate in normal 7H9 media. This can be partially attributed to the idea of uncoupled cellular elongation and division during the growth cycle of mycobacteria. Since the division cycle is governed by time and not cell size/shape, such a defect may not have a direct inhibitory effect on the division and elongation process (30). On this note, the continued growth seen for ΔFtsX in the absence of normal elongation and division is consistent with the depletion of cell division components such as hydrolases in several other bacterial species. For instance, in *E. coli*, neither single nor double mutations in any of the three amidases, AmiA, B, and C, affected the growth rate (20). Nevertheless, these observations make us question how normal growth continues to occur when cells form chains or when they fail to elongate properly.

Supporting evidence has also shown that interrupting amidase activity and disrupting the interaction with FtsX under low osmolarity resulted in the formation of filamented cells in *E. coli* (26). The same effect was seen for mycobacterial ΔFtsE, ΔFtsX, ΔFtsEX, and ΔRipC in addition to the other phenotypes that included bulging, visible chaining, and cell membrane invagination. The type of unusual bulging phenotype displayed by FtsEX could be due to failure in cell pole maturation (31). Most of the cells are shorter and bulgy in this condition, indicative of possible defects in elongation. The low proportion of mutant cells that were in the form of filaments had membrane invaginations, indicating that there might be a significant stall in division. This led to our assumption that FtsEX might have a possible role in facilitating proper septum cleavage. Moreover, cells tend to form long chains when PG hydrolases are not activated, so they continue to elongate without cell division, which is evident in our findings (11, 32).

In addition to their requirement against low osmotic stress, our results showed that FtsEX and RipC seem to be conditionally essential in relation to antibiotic stress. WT cells were undamaged and intact, but the majority of the mutants had holes in their walls. There is a slight similarity between these defects and “open holes,” described as craters, when cells of *S. aureus* were exposed to antimicrobial peptides at sub- and supra-MIC’s in salt-free media (33). The holes could be as a result of membrane rupture, which can be due to the loss of cell wall integrity (34). Another striking observation was the localization of these holes. We noted that they were found to be localized near the poles, which was also observed as “dented spots” in *B. subtilis* after treatment with chlorhexidine (35). The same damaged trait appeared all over cells of *E. coli*; however, the localized morphological damages may be a division scar appearing next to the new pole after completion of a septum and daughter cell separation. Overall, our results show that both mycobacterial FtsE, FtsE, and RipC are conditionally essential for survival in low osmotic stress and during rifampicin treatment. The scanning electronic microscopy imaging also confirms the requirement of the FtsEX-RipC complex for the maintenance of normal mycobacterial morphology in these stressful environments.

## SUPPLEMENTAL MATERIAL

Supplemental Figure S1 spectrum.02515-23-s0001.tiff ΔFtsE, ΔFtsX, ΔFtsEX, and ΔRipC have morphological defects in stressful conditions.

Supplemental Figure S2 spectrum.02515-23-s0002.tiff *M. smegmatis* FtsEX and RipC deletion results in impaired growth in low osmotic stress.

Supplemental Table S1 spectrum.02515-23-s0003.docx MICs of rifampicin for *M. smegmatis* individual strains used in the study.

ASM does not own the copyrights to Supplemental Material that may be linked to, or accessed through, an article. The authors have granted ASM a non-exclusive, world-wide license to publish the Supplemental Material files. Please contact the corresponding author directly for reuse.

## References

[1] World Health Organisation. 2022. World health Organisation tuberculosis fact sheet No.104. WHO, Geneva.

[2] Barter DM, Agboola SO, Murray MB, Bärnighausen T. 2012. Tuberculosis and poverty: the contribution of patient costs in sub-Saharan Africa – a systematic review. BMC Public Health 12:980–980. doi:10.1186/1471-2458-12-980 23150901 PMC3570447

[3] Flynn JL, Chan J. 2003. Immune evasion by Mycobacterium tuberculosis: living with the enemy. Curr Opin Immunol 15:450–455. doi:10.1016/s0952-7915(03)00075-x 12900278

[4] Queval CJ, Brosch R, Simeone R. 2017. The macrophage: a disputed fortress in the battle against Mycobacterium tuberculosis. Front Microbiol 8:2284. doi:10.3389/fmicb.2017.02284 29218036 PMC5703847

[5] Bhat KH, Yaseen I. 2018. Mycobacterium - research and development

[6] Ehrt S, Schnappinger D. 2009. Mycobacterial survival strategies in the phagosome: defence against host stresses. Cell Microbiol 11:1170–1178. doi:10.1111/j.1462-5822.2009.01335.x 19438516 PMC3170014

[7] Hett EC, Rubin EJ. 2008. Bacterial growth and cell division: a mycobacterial perspective. Microbiol Mol Biol Rev 72:126–156. doi:10.1128/MMBR.00028-07 18322037 PMC2268284

[8] Ultee E, Ramijan K, Dame RT, Briegel A, Claessen D. 2019. Stress-induced adaptive morphogenesis in bacteria. Adv Microb Physiol 74:97–141. doi:10.1016/bs.ampbs.2019.02.001 31126537

[9] Zhang YJ, Reddy MC, Ioerger TR, Rothchild AC, Dartois V, Schuster BM, Trauner A, Wallis D, Galaviz S, Huttenhower C, Sacchettini JC, Behar SM, Rubin EJ. 2013. Tryptophan biosynthesis protects mycobacteria from CD4 T-cell-mediated killing. Cell 155:1296–1308. doi:10.1016/j.cell.2013.10.045 24315099 PMC3902092

[10] Aertsen A, Michiels CW. 2004. Stress and how bacteria cope with death and survival. Crit Rev Microbiol 30:263–273. doi:10.1080/10408410490884757 15646400

[11] Mavrici D, Marakalala MJ, Holton JM, Prigozhin DM, Gee CL, Zhang YJ, Rubin EJ, Alber T. 2014. Mycobacterium tuberculosis FtsX extracellular domain activates the peptidoglycan hydrolase. Proc Natl Acad Sci U S A 111:8037–8042. doi:10.1073/pnas.1321812111 24843173 PMC4050617

[12] Kieser KJ, Rubin EJ. 2014. How sisters grow apart: mycobacterial growth and division. Nat Rev Microbiol 12:550–562. doi:10.1038/nrmicro3299 24998739 PMC6556109

[13] Senzani S, Li D, Bhaskar A, Ealand C, Chang J, Rimal B, Liu C, Joon Kim S, Dhar N, Kana B. 2017. An Amidase_3 domain-containing N-acetylmuramyl-L-alanine amidase is required for mycobacterial cell division. Sci Rep 7:1140. doi:10.1038/s41598-017-01184-7 28442758 PMC5430687

[14] Chao MC, Kieser KJ, Minami S, Mavrici D, Aldridge BB, Fortune SM, Alber T, Rubin EJ. 2013. Protein complexes and proteolytic activation of the cell wall hydrolase RipA regulate septal resolution in mycobacteria. PLoS Pathog 9:e1003197. doi:10.1371/journal.ppat.1003197 23468634 PMC3585148

[15] Vermassen A, Leroy S, Talon R, Provot C, Popowska M, Desvaux M. 2019. Cell wall hydrolases in bacteria: insight on the diversity of cell wall amidases, glycosidases and peptidases toward peptidoglycan. Front Microbiol 10:331. doi:10.3389/fmicb.2019.00331 30873139 PMC6403190

[16] Arrigucci R, Pozzi G. 2017. Identification of the chain-dispersing peptidoglycan hydrolase LytB of Streptococcus gordonii. PLoS One 12:e0176117. doi:10.1371/journal.pone.0176117 28414782 PMC5393624

[17] Sham L-T, Barendt SM, Kopecky KE, Winkler ME. 2011. Essential PcsB putative peptidoglycan hydrolase interacts with the essential FtsXSpn cell division protein in Streptococcus pneumoniae D39. Proc Natl Acad Sci U S A 108:E1061–9. doi:10.1073/pnas.1108323108 22006325 PMC3215045

[18] Bajaj R, Bruce KE, Davidson AL, Rued BE, Stauffacher CV, Winkler ME. 2016. Biochemical characterization of essential cell division proteins FtsX and FtsE that mediate peptidoglycan hydrolysis by PcsB in Streptococcus pneumoniae. Microbiologyopen 5:738–752. doi:10.1002/mbo3.366 27167971 PMC5061712

[19] Arends SJR, Kustusch RJ, Weiss DS. 2009. ATP-binding site lesions in FtsE impair cell division. J Bacteriol 191:3772–3784. doi:10.1128/JB.00179-09 19376877 PMC2698383

[20] Heidrich C, Templin MF, Ursinus A, Merdanovic M, Berger J, Schwarz H, de Pedro MA, Höltje JV. 2001. Involvement of N‐acetylmuramyl‐L‐alanine amidases in cell separation and antibiotic‐induced autolysis of Escherichia coli. Mol Microbiol 41:167–178. doi:10.1046/j.1365-2958.2001.02499.x 11454209

[21] Meisner J, Montero Llopis P, Sham L-T, Garner E, Bernhardt TG, Rudner DZ. 2013. Ftsex is required for Cwlo Peptidoglycan Hydrolase activity during cell wall elongation in Bacillus subtilis. Mol Microbiol 89:1069–1083. doi:10.1111/mmi.12330 23855774 PMC3786131

[22] Meier EL, Daitch AK, Yao Q, Bhargava A, Jensen GJ, Goley ED. 2017. FtsEX-mediated regulation of the final stages of cell division reveals morphogenetic plasticity in Caulobacter crescentus. PLoS Genet 13:e1006999. doi:10.1371/journal.pgen.1006999 28886022 PMC5607218

[23] Ricard M, Hirota Y. 1973. Process of cellular division in Escherichia coli: physiological study on thermosensitive mutants defective in cell division. J Bacteriol 116:314–322. doi:10.1128/jb.116.1.314-322.1973 4583216 PMC246424

[24] Schmidt KL, Peterson ND, Kustusch RJ, Wissel MC, Graham B, Phillips GJ, Weiss DS. 2004. A predicted ABC transporter, FtsEX, is needed for cell division in Escherichia coli. J Bacteriol 186:785–793. doi:10.1128/JB.186.3.785-793.2004 14729705 PMC321481

[25] Monahan LG, Hajduk IV, Blaber SP, Charles IG, Harry EJ, Gottesman S. 2014. Coordinating bacterial cell division with nutrient availability: a role for glycolysis. mBio 5:e00935–14. doi:10.1128/mBio.00935-14 24825009 PMC4030479

[26] Reddy M. 2007. Role of Ftsex in cell division of Escherichia coli: viability of ftsEX mutants is dependent on functional sufi or high osmotic strength. J Bacteriol 189:98–108. doi:10.1128/JB.01347-06 17071757 PMC1797223

[27] Zhu J-H, Wang B-W, Pan M, Zeng Y-N, Rego H, Javid B. 2018. Rifampicin can induce antibiotic tolerance in mycobacteria via paradoxical changes in rpoB transcription. Nat Commun 9:4218. doi:10.1038/s41467-018-06667-3 30310059 PMC6181997

[28] Yang DC, Peters NT, Parzych KR, Uehara T, Markovski M, Bernhardt TG. 2011. An ATP-binding cassette transporter-like complex governs cell-wall hydrolysis at the bacterial cytokinetic ring. Proc Natl Acad Sci U S A 108:E1052–60. doi:10.1073/pnas.1107780108 22006326 PMC3215046

[29] Sham L-T, Jensen KR, Bruce KE, Winkler ME. 2013. Involvement of FtsE ATPase and FtsX extracellular loops 1 and 2 in FtsEX-PcsB complex function in cell division of Streptococcus pneumoniae D39. mBio 4:e00431-13. doi:10.1128/mBio.00431-13 23860769 PMC3735124

[30] Aldridge BB, Fernandez-Suarez M, Heller D, Ambravaneswaran V, Irimia D, Toner M, Fortune SM. 2012. Asymmetry and aging of mycobacterial cells lead to variable growth and antibiotic susceptibility. Science 335:100–104. doi:10.1126/science.1216166 22174129 PMC3397429

[31] Claessen D, Emmins R, Hamoen LW, Daniel RA, Errington J, Edwards DH. 2008. Control of the cell elongation–division cycle by shuttling of PBP1 protein in Bacillus subtilis. Mol Microbiol 68:1029–1046. doi:10.1111/j.1365-2958.2008.06210.x 18363795

[32] Yang DC, Blair KM, Salama NR. 2016. Staying in shape: the impact of cell shape on bacterial survival in diverse environments. Microbiol Mol Biol Rev 80:187–203. doi:10.1128/MMBR.00031-15 26864431 PMC4771367

[33] Hartmann M, Berditsch M, Hawecker J, Ardakani MF, Gerthsen D, Ulrich AS. 2010. Damage of the bacterial cell envelope by antimicrobial peptides Gramicidin S and PGLa as revealed by transmission and scanning electron microscopy. Antimicrob Agents Chemother 54:3132–3142. doi:10.1128/AAC.00124-10 20530225 PMC2916356

[34] Biswas D, Tiwari M, Tiwari V. 2019. Molecular mechanism of antimicrobial activity of chlorhexidine against carbapenem-resistant Acinetobacter baumannii. PLoS One 14:e0224107. doi:10.1371/journal.pone.0224107 31661500 PMC6818764

[35] Cheung H-Y, Wong MM-K, Cheung S-H, Liang LY, Lam Y-W, Chiu S-K. 2012. Differential actions of chlorhexidine on the cell wall of Bacillus subtilis and Escherichia coli. PLoS One 7:e36659. doi:10.1371/journal.pone.0036659 22606280 PMC3350502

