# Supplementary figures and images for: Mycobacterial FtsEX-RipC interaction is required for normal growth and cell morphology in rifampicin and low ionic strength conditions
Source: Microbiol Spectr. 2024 Jan 30;12(3):e02515-23. doi: 10.1128/spectrum.02515-23 (PMC10913748; doi:10.1128/spectrum.02515-23)

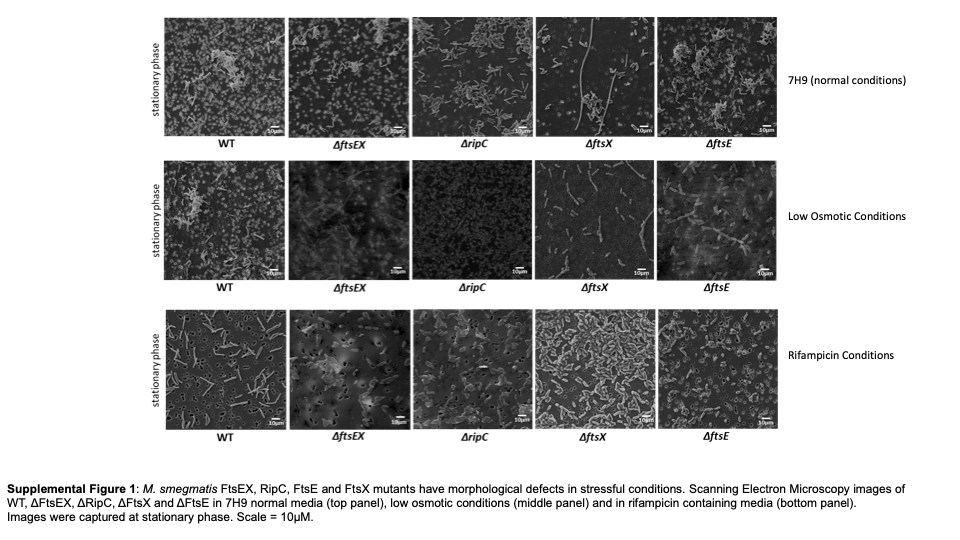

Supplement: Supplemental Figure S1 — ΔFtsE, ΔFtsX, ΔFtsEX, and ΔRipC have morphological defects in stressful conditions. [file spectrum.02515-23-s0001.tiff]

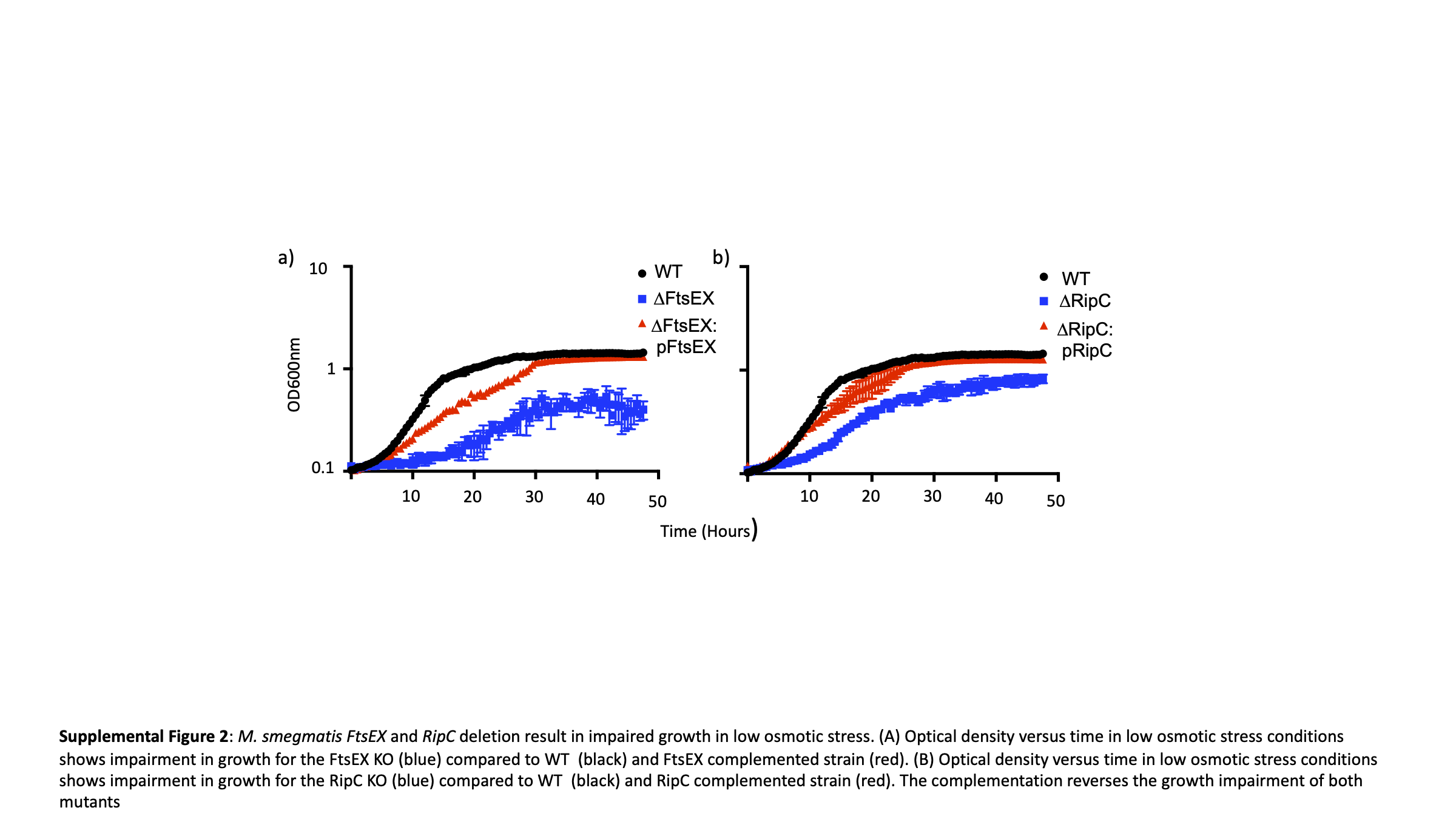

Supplement: Supplemental Figure S2 — M. smegmatis FtsEX and RipC deletion results in impaired growth in low osmotic stress. [file spectrum.02515-23-s0002.tiff]
