# Supplementary material for: Mycobacterial FtsEX-RipC interaction is required for normal growth and cell morphology in rifampicin and low ionic strength conditions
Source: Microbiol Spectr. 2024 Jan 30;12(3):e02515-23. doi: 10.1128/spectrum.02515-23 (PMC10913748; doi:10.1128/spectrum.02515-23)
Supplement: Supplemental Table S1 — MICs of rifampicin for M. smegmatis individual strains used in the study. [file spectrum.02515-23-s0003.docx]

**Supplemental Table 1: Minimum Inhibitory concentrations (MICS) of Rifampicin for indicated *M. smegmatis* strains**

| **Strain:** | WT | ΔFtsEX | ΔFtsX | ΔFtsE | ΔFtsEX:  pFtsEX | ΔFtsEX:  pFtsEX_F61A_ | ΔFtsEX:  pFtsEX_F110A_ | ΔFtsEX:  pFtsEX_Y113A_ | ΔFtsEX:  pFtsEX_F122A_ | ΔRipC | ΔRipC:  pRipC |
| --- | --- | --- | --- | --- | --- | --- | --- | --- | --- | --- | --- |
| **MIC** (mg/ml) | 8 | 1 | 1 | 1 | 8 | 4 | 2 | 2 | 1 | 2 | 8 |
